# A rare case of odontogenic keratocyst extending into the sphenoid bone from the maxilla

**DOI:** 10.1016/j.ijscr.2020.05.003

**Published:** 2020-05-21

**Authors:** Mitsuo Goto, Sei Ueda, Satoru Miyabe, Satoshi Watanabe, Yoshihiko Sugita, Toru Nagao

**Affiliations:** a Department of Maxillofacial Surgery, Aichi Gakuin University, School of Dentistry, 2-11 Suemori-dori Chikusa-ku, Nagoya 464-8651, Japan; b Department of Oral Pathology, Aichi Gakuin University, School of Dentistry, 1-100 Kusumoto-cho Chikusa-ku, Nagoya 464-8650, Japan

**Keywords:** Odontogenic keratocyst, Maxillary sinus, Sphenoid bone, Skull base, Recurrence, Case report

## Abstract

**Background:**

Odontogenic keratocyst (OKC) is the third most common odontogenic cyst which arises from cell rests of dental lamina, and usually observed in the jaws. Because OKC is noted for its high rate of recurrence, there are various treatment strategies. Here, we present a rare case of OKC which occupied the entire maxillary sinus and pterygoid process of the sphenoid bone extending nearly to the skull base.

**Case presentation:**

The patient was a 21-year-old male and underwent surgical removal of the cyst using the Caldwell-Luc procedure which in this case extended the surgical approach to the pterygoid process of the sphenoid bone via the pterygomaxillary junction. However, we found a recurrent lesion in the posterior wall of the maxillary sinus 20 months after the surgery and subsequently performed a secondary cystectomy. Surgical specimens showed positive bcl-2 staining of OKC and negative cytokeratin-10 on immunohistochemistry for both primary and recurrent lesions.

**Conclusion:**

OKC rarely occurs in the maxillary sinus and extends to the deep maxillary structure and the skull base. In order to prevent recurrence, it is necessary to recognize the exact location of the entire lesion. Careful examination of preoperative CT images is needed to make a complete surgical planning and to perform a reliable surgical procedure.

## Highlights

• Odontogenic keratocyst (OKC) is the third most common odontogenic cyst which arises from cell rests of dental lamina.
• Because of high rate of recurrence, there are various treatment strategies.
• OKC is usually observed in the jaws.
• We report on a rare case of OKC which involved the maxillary sinus extending into the sphenoid bone nearly to the skull base.

## 1 Introduction

Odontogenic keratocyst (OKC) is the third most common odontogenic cyst which arises from cell rests of dental lamina. This lesion used to be classified as an odontogenic tumor by the World Health Organization but was reclassified as a cyst in 2017. Because OKC is noted for its high rate of recurrence, there are various treatment strategies for the lesion. Previous studies have reported that enucleation had a recurrence rate of 56%, whereas resection had a rate of 0% but with increased morbidity for the patient [[1], [2], [3]]. Other treatment options for OKC include decompression which is the method taken to reduce the pressure from within a cyst and marsupialization which is the technique based on the externalization of the cyst creating a surgical window [4,5]. Both techniques allow to decrease the size of the cyst, but later require a secondary cystectomy or enucleation. Adjuvant therapy with topical appreciation of Carnoy’s solution, which composed of ferric chloride dissolved in absolute alcohol and glacial acetic acid with or without chloroform, or 5-FU are also attempted after enucleation showing a favorable outcome [3,6].

The most frequently involved site of OKC is the mandibular bone occurring at a rate of around 73% compared with 27% for the maxillary bone [7,8]. Previous reports have indicated that OKC involvement of the maxillary sinus occurred with less than 1% of OKC cases [9]. Moreover, the skull base extension of the lesion is reportedly quite rare [10].

Here, we report on a rare case of OKC which entirely involved the maxillary sinus extending into the pterygoid process of the sphenoid bone nearly to the skull base. This work has been reported in line with the SCARE criteria [11].

## 2 Presentation of case

**Fig. 1:**
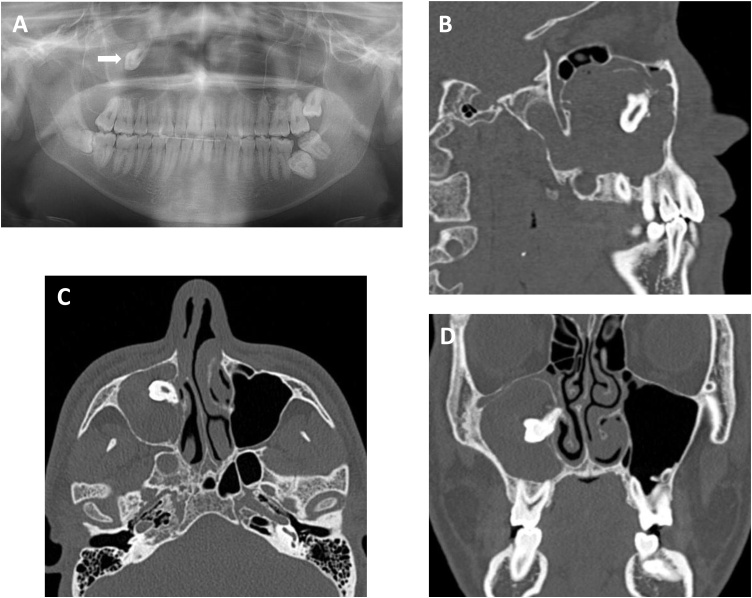
(A) Panoramic radiograph showing a dislocated third molar in the right maxillary sinus (arrow). (B) Sagittal CT scan shows OKC in the right maxillary sinus involving the third molar which has extended into the pterygoid process of the sphenoid bone near the skull base. (C) Axial CT scan. (D) Coronal CT scan.

A 21-year-old male patient was referred to our university hospital to examine his upper right third molar which was displaced into the maxillary sinus based on panoramic radiography. He had no symptoms and no previous medical history. Extraoral examination showed no evidence of facial swelling or asymmetry. On intraoral examination, no abnormal findings were observed. Panoramic radiography showed an ill-defined and radiopaque lesion in the right maxillary sinus involving the third molar that had been displaced to the roof of the sinus (Fig. 1A). Computed tomography (CT) scan revealed an irregularly dense cystic lesion that entirely occupied the right maxillary sinus extending to the right pterygoid process of the sphenoid bone near the skull base (Fig. 1B to 1D).

**Fig. 2:**
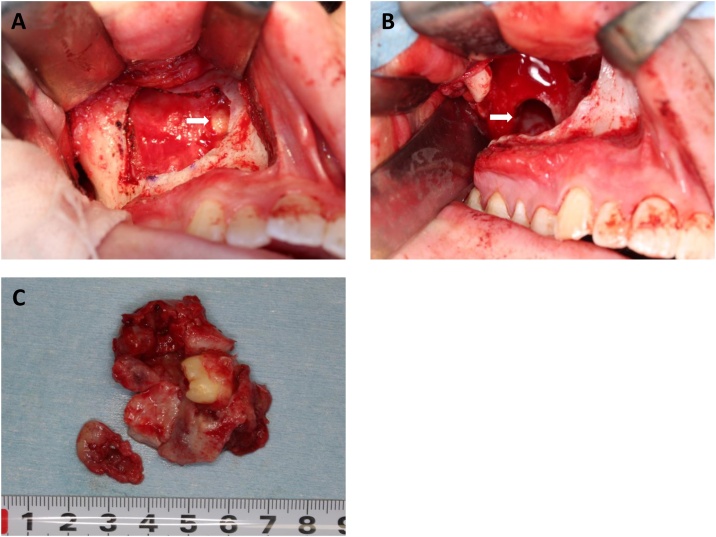
(A) Intraoperative image after removal of the anterior wall of the maxillary sinus showing OKC involvement with a third molar (arrow). (B) Postoperative image showing a bone window to access into the pterygoid process (arrow). (C) Surgical specimen of OKC with the third molar. A larger piece of the lesion was in the maxillary sinus and a smaller one in the pterygoid process.

**Fig. 3:**
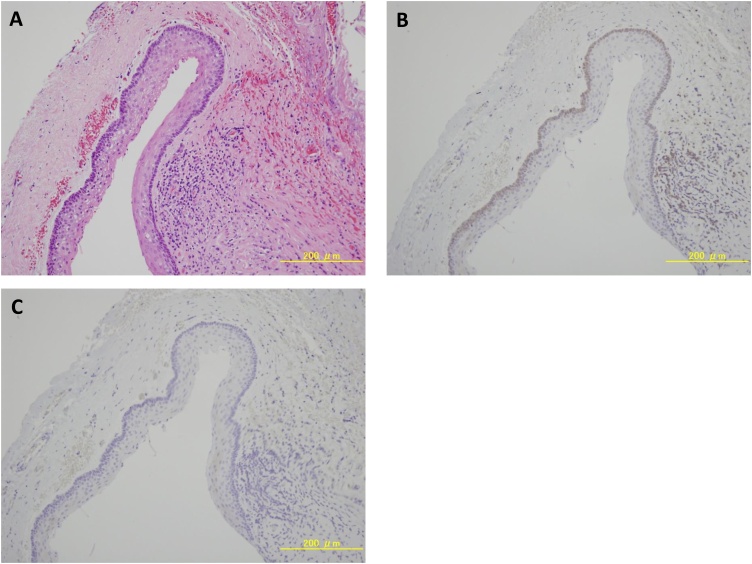
H & E stain and immunohistochemistry for bcl-2 and cytokeratin-10 of the surgical specimen. (A) H & E stain reveals the specimen to be OKC (original magnification ×200). (B) Immunohistochmical staining shows bcl-2 expression in the basal layer (original magnification ×200). (C) No staining is observed for cytokeratin-10 (original magnification ×200).

**Fig. 4:**
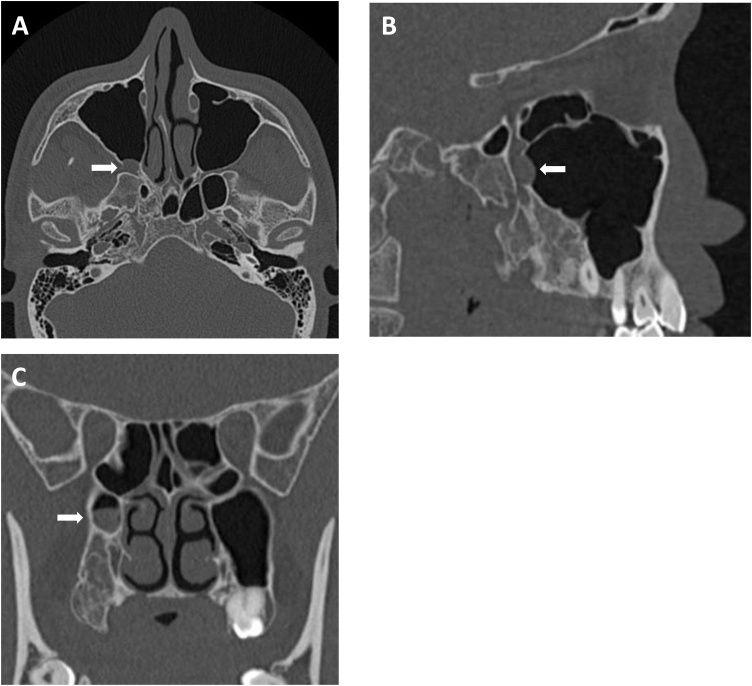
CT scans show recurrent OKC in the posterior wall of the right maxillary sinus (arrows). (A) Axial scan. (B) Sagittal scan. (C) Coronal scan.

**Fig. 5:**
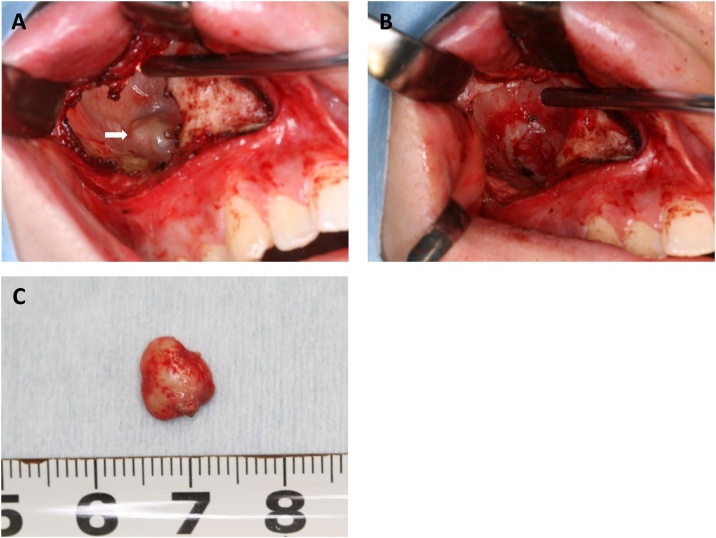
(A) Intraoperative image showing the recurrent lesion in the posterior wall of the right maxillary sinus (arrow). (B) Postoperative image after removal. (C) Surgical specimen of recurrent OKC.

**Fig. 6:**
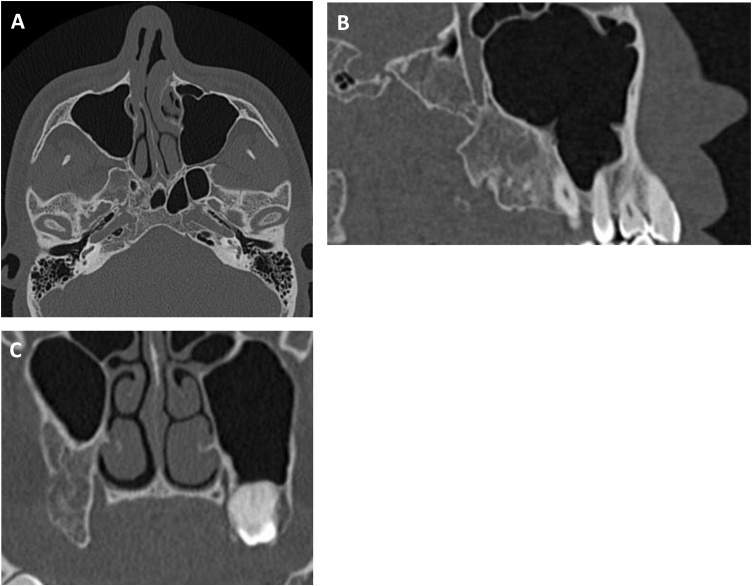
Follow-up CT scans show no recurrent lesion after a secondary cystectomy. (A) Axial scan. (B) Sagittal scan. (C) Coronal scan.

The patient underwent biopsy and histopathological examination diagnosed the specimen to be OKC. We performed enucleation of the cyst and removal of the displaced third molar using the Caldwell-Luc procedure which in this case extended the surgical approach to the right pterygoid process of the sphenoid bone via the pterygomaxillary junction (Fig. 2A to 2C). Histopathological examination of the surgical specimen showed that the cyst wall consisted of fibrous tissue with mild chronic inflammatory cell infiltration, and a stratified squamous epithelium with parakeratinization was observed. The boundary between the epithelium and connective tissue was flat and a palisade arrangement was observed in the epithelial basal cells (Fig. 3A). Based on these features, we confirmed the lesion to be OKC. Immunohistochemistry for bcl-2 showed limited expression of the basal layer of OKC lining whereas no expression was found for cytokeratin-10 staining (Fig. 3B and 3C). After surgery, a careful follow-up was done. However, we found a recurrent lesion in the posterior wall of the maxillary sinus on CT scan 20 months after the surgery (Fig. 4A to 4C). The patient subsequently underwent a secondary cystectomy (Fig. 5A to 5C) and histopathological examination confirmed the specimen to be OKC. Regarding immunohistochemistry, we found positive bcl-2 staining in the basal layer of OKC and negative cytokeratin-10 which was the same pattern as the primary specimen. No evidence of recurrence has been observed over the second postoperative period of 20 months (Fig. 6A to 6C).

## 3 Discussion

In the study of odontogenic cysts patients, OKC is reportedly diagnosed at 19.1% [12]. Previous studies have shown that OKC commonly affected patients in their second and third decades of life, and occurred more frequently in men [13]. In our case, the patient was a 21-year-old man who was exactly in line within these ranges, and the location of the lesion was in the entire maxillary sinus extending into the pterygoid process of the sphenoid bone. A previous study has confirmed that the most common OKC site is the posterior mandible at a rate of 45% followed by the maxillary molar and tuberosity region at 22.5% [7]. Another study has indicated that the canine region was the most common location for OKC in the maxilla [14]. As is the current case, maxillary sinus involvement of OKC with an ectopic tooth is very rare [15]. Moreover, the current case uncommonly developed into the pterygoid process across the pterygomaxillary junction invading nearly to the skull base. Regarding the original site of the lesion, we supposed that it would occur in the maxillary tuberosity region where the third molar had impacted, and then the OKC had kept extending resulting in displacement of the third molar tooth bud. We also supposed that this pathologic condition perforated the adjacent posterior wall of the maxillary sinus and then extended into the pterygoid process involving a part of the sphenoid sinus. Interestingly, the affected side of the sphenoid sinus was less developed compared with the other side according to the CT scan. Previous studies have shown that development of the sphenoid sinus is completed past the age of ten [16]. An imaging study has also identified that the sphenoid sinus attains its mature size by the age of 14 years [17]. These studies may indicate that the sphenoid bone lesion in our case may have occurred around the age of 14.

There have been many arguments made regarding the best treatment for OKC, and it is still controversial because the most significant features of OKC is its potentially aggressive behavior, tendency to recur, and neoplastic potential compared with other jaw cysts. Treatment options for OKC include aggressive curettage, enucleation with or without Carnoy’s solution or 5-FU, marsupialization, and resection [1,3]. One of the advantages to using marsupialization is not only that it decreases the size of the cyst but also that marsupialization results in epithelial differentiation changing expression of cytokeratin-10 from positive to negative, which also returns the cyst lining to a more normal oral epithelium [5,18]. In the present case, we first considered to perform marsupialization expecting volume reduction of the lesion. However, if osteogenesis were to occur around the pterygomaxillary junction after marsupialization, the pterygoid process lesion would become isolated from the maxillary sinus lesion. In order to keep an open surgical route to the pterygoid process lesion from maxillary sinus, we decided to employ enucleation instead. This surgical approach via the maxillary sinus seemed the easiest way to remove the pterygoid process lesion without the traumatic morbidity caused by bone removal and unexpected bleeding. We did not employ Carnoy’s solution in the surgical wound after removal of the lesion. Because the use for the thin bony wall of the maxillary sinus and the orbit may cause necrosis of these structures. We also concerned the possible complication in the middle cranial fossa resulting from the use for the sphenoid bone.

Regarding immunohistochemistry, the present case showed negative staining of cytokeratin-10 and positive of bcl-2. Previous reports have indicated that bcl-2 protein was consistently expressed in the basal cells of OKC but was not expressed in other odontogenic cysts or normal oral mucosa [4,19]. Because bcl-2 is an antiapoptotic protein, positive expression pattern in OKC would represent an aggressive growth pattern of the lesion.

The current case had a recurrence in the posterior wall of the maxillary sinus 20 months after initial surgery. A systematic review of the literature has identified that enucleation with adjunctive therapy, such as Carnoy’s solution application or decompression before enucleation, had a recurrence rate of 1%–8.7%, whereas simple enucleation had a rate of 17%–56% [1]. Kinard et al. reported a mean recurrence time of 22.3 months after enucleation with or without adjunctive therapy or decompression with or without secondary cystectomy [20]. The reason for OKC recurrence has been attributed to several mechanisms including a high proliferative potential, histological features of daughter cysts, and incomplete removal at primary surgery [21]. Also, patients with basal cell nevus syndrome have a high incidence of daughter or satellite cysts making complete removal more complex [2]. The current case did not have any syndrome. But after careful review of the CT scan, we confirmed that a recurrent lesion occurred from a small satellite cyst that we had left behind after initial cystectomy. Kinard et al. also reported that multilocular OKC lesions were 33.6 times more likely to recur than unilocular lesions [20]. Careful examination of preoperative CT images is needed to make a complete surgical planning and to perform a reliable surgical procedure.

## 4 Conclusion

In conclusion, OKC rarely occurs in the maxillary sinus and extends to the deep maxillary structure and the skull base. In order to prevent recurrence, it is necessary to recognize the exact location of the entire lesion. Careful follow-up is also needed to detect recurrence especially in the deep maxillary structure.

## Funding

This case report did not receive any specific grant from funding agencies in the public, commercial, or not-for-profit sectors.

## Ethical approval

Ethical approval is exempted as this is a case report.

## Consent

Written informed consent was obtained from the patient for publication of this case report and accompanying images.

## Author contribution

Mitsuo Goto: case report design, writing the manuscript.

Sei Ueda: data collection, manuscript preparation.

Satoru Miyabe: followed up the patient, contributed to the pathology section.

Satoshi Watanabe: followed up the patient, reviewed the manuscript.

Yoshihiko Sugita: contributed to the pathology section, literature search.

Toru Nagao: reviewed the manuscript, revised the manuscript.

## Registration of research studies

Not applicable.

## Research studies

Name of the registry: Not applicable.

Unique identifying number or registration ID: Not applicable.

Hyperlink to your specific registration (must be publicly accessible and will be checked): Not applicable.

## Guarantor

Mitsuo Goto.

## Provenance and peer review

Not commissioned, externally peer-reviewed.

## Declaration of Competing Interest

The authors declare no conflicts of interest.
